# Free Will, Determinism, and Epiphenomenalism

**DOI:** 10.3389/fpsyg.2018.02623

**Published:** 2019-01-09

**Authors:** Mark Balaguer

**Affiliations:** Department of Philosophy, California State University, Los Angeles, CA, United States

**Keywords:** free will, determinism, epiphenomenalism, Libertarianism, torn decisions, non-randomness

## Abstract

This paper articulates a non-epiphenomenal, libertarian kind of free will—a kind of free will that's incompatible with both determinism and epiphenomenalism—and responds to scientific arguments against the existence of this sort of freedom. In other words, the paper argues that we don't have any good empirical scientific reason to believe that human beings don't possess a non-epiphenomenal, libertarian sort of free will.

## 1. Introduction

There's a very old, very traditional argument against free will that's based on the claim that (D1) our decisions are causally determined (or for-all-practical-purposes causally determined, or some such thing) by prior events, and (D2) this is incompatible with free will. We can think of this as the *backward*-looking problem of free will because it has to do with the causal antecedents of our decisions. There's a much more recent argument against free will that's *forward*-looking, or to put the point differently, that arises out of the thought that some sort of *epiphenomenalism* is true, rather than the thought that some sort of *determinism* is true. The worry might be put like this: (E1) our decisions aren't the causes of our actions (i.e., our decisions are *epiphenomenal*), and (E2) this is incompatible with free will.

You might think that we should respond to the first of these arguments by rejecting (D2). I won't be concerned with such responses here. This isn't because I'm convinced that (D2) is true; it's because I think it doesn't really matter whether it's true. I've argued for this stance elsewhere (Balaguer, 2010, 2016) and won't rehearse the argument here. Briefly, though, the thought is as follows: (a) we can easily define some kinds of freedom that are compatible with determinism; and (b) we can easily define some kinds of freedom that are incompatible with determinism; and (c) the question of whether free will—i.e., *real* free will— is compatible with determinism boils down to the question “Which of the various kinds of freedom that we can define is real free will?”; and (d) this latter question is a purely semantic question.

Rather than bogging down in the semantic question of what free will is, I'm going to stipulatively define a variety of freedom that's incompatible with determinism—and also with epiphenomenalism^1^—and I'm going to focus on the question of whether we have *that* kind of freedom, i.e., the kind that's indeterministic and non-epiphenomenal by *definition*. Here's an initial, rough characterization of the sort of freedom I've got in mind:

*NEL-Freedom (initial, rough definition)*: A person is *non-epiphenomenal, libertarian free* (or for short, *NEL-free*) if and only if she makes at least some decisions that are both undetermined (in a libertarian sort of way—more on what this means later) and non-epiphenomenal (i.e., that play an appropriate role in the causation of our actions—again, more on what this means later).

Let *NE-libertarianism* be the view that human beings are NEL-free. My aim in this paper is to defend this view against recent anti-free-will arguments that proceed by trying to motivate claims like (D1) and (E1). The arguments I'll be responding to are based on empirical scientific findings. Thus, in essence, what I'm going to be arguing is that the scientific arguments that have arisen in recent years against the existence of free will—the arguments that proceed by trying to (empirically) motivate the claim that our decisions are epiphenomenal and/or causally determined (or for-all-practical-purposes determined)—are not good arguments.

I should say here that I'll be assuming that mind-brain materialism is true; in particular, I'll assume that our decisions are physical events, presumably neural events. It follows pretty quickly from this that the relevant kinds of determinism and epiphenomenalism—i.e., (D1) and (E1)—are empirical claims. But if this is right, and if I'm right that we don't have any good empirical-scientific reason to endorse (D1) or (E1), then I think it can be argued pretty quickly that we don't have *any* good reason to believe (D1) or (E1)—i.e., that we don't have any good reason to think that our decisions are causally determined or epiphenomenal in ways that would be incompatible with the sort of freedom that I'll be defining in this paper.

I, of course, can't respond here to *every* empirical-science-based argument for (D1) and (E1). I'll focus on arguments based on results from psychology and neuroscience. In connection with (E1), these are pretty obviously the most important arguments, but in connection with (D1), you might doubt that the most important results come from psychology and neuroscience; for you might think we have good reason to endorse some deterministic interpretation of quantum mechanics—and, hence, good reason to endorse universal determinism. I argued in Balaguer (2010) that we in fact don't have good reason to endorse any specific interpretation of quantum mechanics—deterministic or indeterministic—and that, because of this, we don't have any good reason to endorse universal determinism. I also argued there that we don't have any reason to believe that all *neural* events are determined. I can't rehearse the arguments for these claims here, but if they're right, then the question we should be focused on, vis-à-vis (D1), is the very specific question of whether our *decisions* are determined in some freedom-undermining way. And the places to look for evidence for the claim that our decisions *are* determined in some such way are presumably psychology and neuroscience.

In section 2, I'll list some reasons (based on findings from psychology and neuroscience) for thinking that (D1) and (E1) are true—and, hence, for doubting that we have free will (or, at any rate, NEL-freedom). In section 3, I'll provide a much more careful characterization of NEL-freedom—i.e., the kind of freedom that I'll be defending against the anti-free-will considerations of section 2. And in section 4, I'll respond to those anti-free-will considerations—i.e., I'll argue that they don't give us any good reason to doubt that human beings are NEL-free.

## 2. Worries About Free Will

A lot of studies have been done by psychologists and neuroscientists that raise doubts—both backward-looking determinism-based doubts and forward-looking epiphenomenalism-based doubts—about the hypothesis that human beings have free will. Some of the prominent forward-looking considerations are as follows:

F1. Consciousness is sluggish. In particular, conscious awareness of certain kinds of actions and processes comes *after* the occurrences of the actions and processes themselves (see e.g., Velmans, 1991; Wegner, 2002). Consider, e.g., the processing of incoming speech and quick reactions in emergency situations (e.g., when a driver yanks her steering wheel to the side to avoid hitting someone who has stepped in front of her car). There's reason to think that we only become aware of preforming these actions *after* we perform them; and this suggests that they're not under our conscious control.
F2. People often don't know why they perform certain actions, and they confabulate reasons for their actions—i.e., they construct false theories of why they perform certain actions, seemingly without knowing that the theories are false. There is a lot of evidence for this; see e.g., Festinger (1957), and for interesting split-brain studies related to this, see Gazzaniga (1983).
F3. We're often completely unaware of why we perform certain actions, and we have to infer what our reasons were from our behavior—in the same way that we infer what other people's reasons are from their behavior (see e.g., Nisbett and Wilson, 1977).
F4. While it's true that we experience our decisions, we don't experience our decisions causing our actions. We have to *infer* that our decisions cause our actions from the fact that they precede our actions.
F5. We can be duped into thinking that we willed certain kinds of actions (or caused certain kinds of bodily movements, e.g., hand movements) that were in fact performed by someone else (see e.g., Nielson, 1963; Wegner and Wheatley, 1999). Moreover, we can be duped into thinking that we *didn't* perform certain kinds of actions that we in fact did perform—consider, e.g., the experiences that some people have with Ouija boards.

These results seem to fit very poorly with the hypothesis that human beings have conscious control over their actions. And, more generally, they fit poorly with the view that we intuitively have of ourselves as being something approaching ideal agents—i.e., agents who (a) have reasons for actions, and (b) weigh those reasons against one another in deliberation, and (c) consciously decide what to do, based on our deliberations, in ways that guide our behavior.

Some of the prominent backward-looking (i.e., determinism-based) anti-free-will considerations are as follows:

B1. Our decisions and actions are often causally influenced by unconscious mental states (or, more precisely, events involving us having unconscious mental states) and brain processes that we're not aware of. (This is virtually undeniable; if we've learned anything in empirical psychology over the last hundred years or so, it's that this is true; and this, of course, raises worries about whether our actions are under our conscious control.)
B2. Our decisions and actions are often influenced by situational factors like mood that, intuitively, seem unimportant (see e.g., Milgram, 1969, and for a discussion, see Nelkin, 2005).
B3. Conscious choices can be causally influenced by magnetic stimulation to the brain. In a study done by Brasil-Neto (1992), subjects had to choose between raising their left fingers and raising their right fingers, and their choices were correlated with whether their brains were magnetically stimulated on the left side or the right side.
B4. Conscious decisions are preceded in the brain by non-conscious neural processes that seem (or at any rate, have seemed to some) to be part of the mechanism that actually causes our actions (see e.g., Libet et al., 1983).
B5. There are neural processes that precede our conscious decisions by as much as 7–10 s that can be used to predict which options we'll choose in certain kinds of decisions. To say a bit more, in recent studies performed by Haynes (2011), subjects were given two buttons, one for their left hand and one for their right, and they were told to make a decision at some point as to whether to press the left button or the right button and to then go ahead and push the given button. Using fMRI, Haynes found unconscious brain activity that predicted whether subjects would press the left button or the right; moreover, he found that this activity arose 7–10 s before the person made the conscious decision to push the given button.

These results are compatible with the non-epiphenomenal hypothesis that our decisions cause our actions; but they seem to imply that our decisions are caused by prior events in ways that are incompatible with the hypothesis that human beings have a traditional, libertarian sort of free will.

(You might think that B4 and B5 are backward-looking *and* forward-looking—i.e., that they motivate some sort of epiphenomenalism as well as some sort of determinism. I don't think this is true; for it could be that (a) the mechanisms that cause our actions start running before we consciously decide to perform those actions, and (b) these mechanism go *through* our conscious decisions. But it doesn't matter whether I'm right about this; for if the responses that I'll give in section 4 to B4-B5-style worries about determinism are right, then they'll bring with them responses to B4-B5-style worries about epiphenomenalism as well).

## 3. Ne-Libertarianism

Taken together, considerations F1-F5 and B1-B5 might seem to provide powerful evidence for the claim that human beings don't have free will and, in particular, that they don't have libertarian freedom. But I think these appearances are deceiving. In this section, I'll characterize a kind of non-epiphenomenal libertarian freedom—namely, *NEL-freedom*—and in section 4, I'll argue that the considerations that I just listed in section 2 don't give us any good reason to doubt that human beings are NEL-free. I'll proceed somewhat slowly in this section, getting into the details of the NE-libertarian view—i.e., the view that humans beings are NEL-free. This is because we'll need to have these details in place in order to see why considerations F1-F5 and B1-B5 don't in fact undermine NE-libertarianism.

I'll start by defining libertarian-freedom (or L-freedom); then I'll define libertarianism in terms of L-freedom; then I'll articulate a specific version of libertarianism that I'll call “thin libertarianism”; then at the end, I'll define NEL-freedom and NE-libertarianism.

### 3.1. L-Freedom

To say that a person is L-free is, for starters, to say that some of her decisions are undetermined—i.e., not causally determined by prior events. But indeterminacy by itself is not enough for L-freedom; for undetermined events can be *random* in ways that are incompatible with the sort of freedom that libertarians have in mind. Thus, we can define L-freedom like this:

A person is *libertarian-free*—or for short, *L-free*—if and only if she makes at least some decisions such that (a) they are undetermined and appropriately non-random, and (b) the indeterminacy is relevant to the appropriate non-randomness in the sense that it *procures* the non-randomness, or *increases* it, or *enhances* it, or some such thing.

More needs to be said about what appropriate non-randomness is. There are various views you might endorse here, but however the details go, we should all agree that the relevant sort of non-randomness consists in a kind of *agent-involvedness*. For example, one might say that it consists in the agent *controlling* which option is chosen, or *authoring* the choice, or being the *source* of the choice, or making a *rational* choice, or some combination of these things. Also, many libertarians would follow Kane (1996) in requiring *plural* control (or authorship or whatever)—i.e., in requiring it to be the case that even if the agent had chosen differently, she still would have controlled it (or authored it, or whatever).

Also, more needs to be said about clause (b) of the above definition. To see why this clause is needed, consider the following view:

*Humeanism with a smidge of irrelevant indeterminism*: Our decisions are caused by our reasons, and so they count as *ours* (i.e., appropriately non-random, under our control, and so on). But our decisions aren't *deterministically* caused by our reasons; there are unimportant quantum indeterminacies buried in our decision-making processes; in particular, the prior-to-choice probabilities of our decisions going the way that they in fact go is always extremely high (0.999999, or whatever) but not 1.

This isn't a libertarian view because the indeterminacy is irrelevant to the freedom of our choices. Libertarians think that indeterminacy is *needed* for freedom—and that's why I've included clause (b) in the definition of L-freedom.

### 3.2. Libertarianism

I'll use the term ‘libertarianism' to denote the view that human beings are L-free. This is a bit non-standard. A more standard definition would take libertarianism to be the view that (i) humans are L-free, and (ii) L-freedom is free will. On this way of proceeding, we could say that thesis (i) is the *metaphysical* half of libertarianism and thesis (ii) is the *semantic* (or *conceptual*) half. But thesis (ii) won't be relevant at all to the arguments of this paper, and so to keep things simple, I'm going to use ‘libertarianism' to denote thesis (i).

(On this usage, libertarianism doesn't entail that free will (as opposed to L-freedom) is incompatibilism with determinism, and it doesn't entail that human beings have free will; indeed, it doesn't entail *anything* about free will. So this is definitely non-standard usage. But no harm will come of this)^2^.

### 3.3. Thin Libertarianism

Thin libertarianism is a specific version of the sort of libertarianism that I just defined. There are five main features of thin libertarianism.

First, thin libertarianism involves a commitment to *mind-brain materialism*. In particular, on this view, conscious decisions are physical events, presumably neural events.

Second, thin libertarianism is an *event-causal* view; in other words, on this view, L-free decisions are non-deterministically caused (or probabilistically caused) by prior events, presumably agent-involving events, e.g., events having to do with the agent's reasons. So, importantly, thin libertarianism doesn't involve any sort of irreducible agent causation.

Third, thin libertarianism does not involve the claim that *all* of our actions are L-free, or even undetermined. We perform a *lot* of actions. Just in the course of a single minute, you might perform twenty actions. Think, for instance, of what you do when you drive somewhere. You get in the car; you put your seatbelt on; you put your key in the ignition; you turn the key; you push your foot down on the gas; you put the car in gear, you look in the mirror; and so on. We're almost constantly doing things. We barely even notice them. And we certainly don't consciously decide to do all of these things. Life would be an unbearable nightmare if we had to consciously decide to do everything we do; we'd have to constantly think thoughts like this: “Move your left foot forward; now your right; left again; right; etc., etc., etc.” We don't want to have to decide to do all of the things we do; we want to be free to think about other things while we're strolling through parks.

The upshot of this, it seems to me, is that the question of free will isn't about the gigantic set of actions we perform; it's about our conscious *choices*, or *decisions*. At any rate, this is what thin libertarianism is about. Indeed, it's really about a certain subset of our conscious decisions, namely, what I've elsewhere (2010) called “*torn decisions.”* We can define torn decisions as follows:

A *torn decision* is a decision in which the person in question has reasons for multiple options, feels torn as to which option is best (and has no conscious belief as to which option is best), and decides without resolving the conflict, i.e., decides while feeling torn.

We seem to make decisions like this many times a day about things like whether to have cereal or yogurt for breakfast, or whether to walk to work or drive, or whatever. But we can also make torn decisions in potentially life-changing situations; e.g., you might have a good job offer in a city you don't like, and you might have a deadline that forces you to decide while feeling utterly torn.

Torn decisions should be distinguished from three other kinds of decisions. First, they should be distinguished from *leaning decisions*; these are decisions in which the agent chooses while leaning toward one of her live options, whereas in a torn decision, the agent feels *completely* torn. Second, torn decisions should be distinguished from *Buridan's-ass decisions*; these are similar to torn decisions except that the various tied-for-best options are more or less indistinguishable, and because of this, the agent *doesn't feel torn*. (For example, if you want a can of tomato soup, and there are ten cans of the same kind on the shelf, you won't feel torn—you'll just grab one and be on your way^3^). Third, torn decisions should be distinguished from what Kane (1996) calls *self-forming actions*, or SFAs. The most important difference here is that whereas SFAs are defined as being undetermined, torn decisions are not. Torn decisions are defined in terms of their phenomenology. So we know from experience that we make some torn decisions—in fact, we make a lot of them—and it's an open empirical question whether some of these decisions are undetermined.

To see why thin libertarianism is about torn decisions, rather than other kinds of decisions, consider the following two decisions:

*Non-Torn Decision* (or for short, *NTD*): You live in a city you hate because you have a job there and can't find another job. You also hate the job in many ways but you keep it because you can't find anything better. You dream of living in City C and having a job at Institution I. Then you're offered a job at institution I, in City C, with a starting salary three times greater than what you presently make. You have to decide whether to accept the offer. All of your reasons favor accepting it, and none of them favor turning it down. *Torn decision* (or for short, *TD)*: You live in your favorite city; you have a job that's OK, but you're not wild about it. You dream of working for Institution I. Then you're offered a job at Institution I, but it's in City C, and you hate City C. You deliberate for a week about whether to take the job, but you still feel completely torn about whether to take the offer, and the deadline is right now, and you have to decide while feeling torn.

It's easy to understand why people would want their torn decisions—decisions like TD—to be undetermined. For one might think that (a) if decisions like TD are determined, then they're determined by things outside of our conscious reasons and thought, and (b) if this is true, then we don't really author and control these decisions, and hence, they aren't fully free. In contrast with this, it's hard to see why anyone would want decisions like NTD to be undetermined. Indeed, it seems to me that we should want decisions like this to be determined by our reasons for action (or, more precisely, by events involving us having the reasons that we have).

In any event, the kind of libertarianism that I'm currently describing—i.e., *thin* libertarianism—is a thesis about torn decisions. Roughly (I'll make this more precise below), it's the thesis that at least some of our torn decisions are L-free (i.e., undetermined, appropriately non-random, and so on).

Simplifying a bit, we can think of a thin-libertarian agent as someone who (a) mostly plods through life in a roughly Humean way—doing things without making conscious decisions, being driven (mostly unconsciously) by reasons for action, not exercising anything like L-freedom—but who (b) comes to a fork in the road every once in a while (sometimes once an hour, sometimes less, sometimes more) and has to make a torn decision about which way to go.

This picture is simplified—e.g., because it ignores leaning decisions—but it gives us a rough idea of what I have in mind. To be clear, though, I do not think that torn decisions are the only kinds of decisions that can be L-free, or that one might reasonably want to be L-free. For example, one might wonder whether our leaning decisions are L-free. But for a variety of reasons, I think that torn decisions are the most important decisions to focus on; indeed, I've argued elsewhere (2010) that human beings are L-free if and only if some their torn decisions are L-free, so that the question of whether we're L-free comes down to the question of whether some of our torn decisions are L-free. But I won't try to argue for this here; I'm just going to focus on torn decisions; and I'm going to take thin libertarianism to say that some of our torn decisions are L-free and to not say anything about any non-torn decisions.

Fourth, note that the claim here is that *some* of our torn decisions are L-free. Libertarianism is perfectly compatible with the claim that some of our torn decisions are causally determined by prior events; e.g., it's compatible with the claim that some of these decisions are determined by subconscious reasons that we're not aware of^4^ All libertarianism says is that *some* of our decisions are undetermined and L-free.

Fifth and finally, it's important to get clear on the kind of indeterminacy that's required for torn decisions to be L-free. This sort of indeterminacy can be defined as follows:

A torn decision is *wholly undetermined* at the moment of choice—or, for short, *TDW-undetermined*—if and only if the actual objective moment-of-choice probabilities of the various reasons-based tied-for-best options being chosen match the phenomenological probabilities—or what the probabilities *seem to us* to be—so that these moment-of-choice probabilities are all more or less even, given the complete state of the universe and all of the laws of nature, and the choice occurs without any other significant causal inputs, i.e., without anything else being causally relevant in a significant way to which option is chosen.

It's important to note that this sort of indeterminacy is compatible with various features of the decision being fully determined. Suppose, e.g., that I'm about to make a torn decision between options A and B. It could be determined that (i) I'm going to make a torn decision (i.e., I'm not going to refrain from choosing), and (ii) I'm going to choose between A and B (i.e., I'm not going to choose some third option that I don't like as much), and (iii) the objective moment-of-choice probabilities of A and B being chosen are both 0.5. All of this is perfectly consistent with the decision being TDW-undetermined. All that needs to be undetermined, in order for the choice to be TDW-undetermined, is *which tied-for-best option is chosen*.

It's also important to note that TDW-indeterminacy lies at one end of a spectrum of possible cases and that there are *degrees* of the kind of indeterminacy I'm talking about here. To see what I've got in mind by this, suppose that Ralph makes a torn decision to order chocolate pie instead of apple pie. Since this is a torn decision, we know that given all of Ralph's conscious reasons and thought, he feels completely neutral between his two tied-for-best options. But it might be that, unbeknownst to Ralph, there are external factors—things that are external to Ralph's conscious reasons and thought (e.g., unconscious mental states, or non-mental brain events that precede the decision)—that causally influence the choice and wholly or partially determine which option is chosen. Indeed, there's a spectrum of possibilities here. At one end of the spectrum, which option is chosen is TDW-undetermined, so that the objective moment-of-choice probabilities of the two tied-for-best options being chosen are 0.5 and 0.5, and nothing else significantly causally influences which option is chosen. At the other end of the spectrum, the choice is fully determined—i.e., factors external to Ralph's conscious reason and thought come in and, unbeknownst to Ralph, cause him to choose chocolate. And in between, there are possible cases where the objective moment-of-choice probabilities are neither 0.5 and 0.5 nor 1 and 0—i.e., where they're 0.8 and 0.2, or 0.7 and 0.3, or whatever; in these cases, external factors causally influence the choice without fully determining it, so that which option is chosen is partially determined and partially undetermined^5^

### 3.4. The Central Libertarian Thesis

In order to fully define thin libertarianism, I need to say a few words about a well-known philosophical argument against libertarianism. The argument I have in mind can be put like this:

*The randomness argument*: Even if our decisions are undetermined in the way that's needed for L-freedom, it doesn't matter because undetermined events are just *random* events. In other words, they occur by *chance*—i.e., they *just happen*. Thus, if we introduce an undetermined event into a decision-making process, that would seem to either (a) increase the level of randomness in that process or (b) leave the level of randomness alone (if the indeterminacy ends up not mattering). So it's hard to see how the introduction of an undetermined event into a decision-making process could increase *non*-randomness. Thus, since this is precisely what's needed for L-freedom, it seems that we don't have L-freedom; indeed, it seems that L-freedom is impossible^6^

I think that libertarians can respond to this argument by arguing for the following thesis:

*Central Libertarian Thesis (CLT)*: If our torn decisions are undetermined in the right way—i.e., if they're TDW-undetermined—then they're appropriately non-random and L-free.

If we take *TDW-indeterminism* to be the view that some of our torn are TDW-undetermined, and if we assume (as I am here—see above) that libertarianism is true if and only if some of our torn decisions are L-free, then CLT can be put more succinctly as follows:

*CLT (alternate formulation):* If TDW-indeterminism is true, then libertarianism is true.

If CLT is true, then it turns the randomness argument completely on its head. The randomness argument says that indeterminacy implies randomness. CLT, on the other hand, says that the right kind of indeterminacy implies *non*-randomness. If this is right (and if I'm right that libertarianism is true if and only if our torn decisions are L-free), then the question of whether libertarianism is true reduces to the purely empirical question of whether TDW-indeterminism is true.

I argued for CLT at length in Balaguer (2010). I can't rehearse all of my arguments here, but I'd like to say a few words about one of them. If indeterminism is true, then there are at least some physical events that are undetermined. These undetermined events are events that determine how the universe will evolve. So, for example, suppose that I'm going to be in an ice cream parlor tonight and that at some specific time—say, 8:00 p.m.—I'm going to make a torn decision about whether to order chocolate or vanilla ice cream. If indeterminism is true—and, in particular, if it's not yet determined whether I'm going to order chocolate or vanilla ice cream—then there's some undetermined event E (or some collection of undetermined events, but let's simplify and suppose that it's a single event) that will occur between now and 8:00pm tonight that will determine whether the universe evolves in an I-get-chocolate-ice-cream way or an I-get-vanilla-ice-cream way. Now notice the following crucial point: if TDW-indeterminism is true, then *E is my torn decision*. In other words, the undetermined physical event that, so to speak, spins the universe off in an I-get-chocolate-ice-cream direction, instead of an I-get-vanilla-ice-cream direction, just *is* my conscious decision—i.e., it's the mental event with a me-choosing-now phenomenology.

This follows straightforwardly from TDW-indeterminism (together with the mind-brain materialist assumption that decisions are physical events)^7^ So if TDW-indeterminism is true, then we get the result that my conscious decision *is* the undetermined physical event that settles whether the universe evolves in an I-get-chocolate-ice-cream way or an I-get-vanilla-ice-cream way. I argued in Balaguer (2010) and Balaguer (in progress) that if this is true—if our torn decisions *are* the undetermined events that settle which of our tied-for-best options get chosen—then (i) our torn decisions are appropriately non-random (e.g., we author and control these decisions in important ways); and (ii) the indeterminacy procures the appropriate non-randomness, so that our torn decisions are also L-free; and (iii) this gives us everything we want, or should want, out of libertarianism. But I can't argue for all of these points here.

### 3.5. Thin Libertarianism Defined

Given everything I've said, we can define thin libertarianism as the view that TDW-indeterminism is true—i.e., that at least some of our torn decisions are TDW-undetermined—and, hence, that at least some of these decisions are appropriately non-random and L-free.

### 3.6. NEL-Freedom

I think that thin libertarianism captures the *backward*-looking claim that libertarians should endorse. But it doesn't make any *forward*-looking claims; in particular, it's compatible with the epiphenomenalist thesis that our torn decisions don't play any role in causing our actions. If libertarians want to avoid this result, then they need to define a kind of libertarian freedom that requires non-epiphenomenalism. We can do this as follows:

A person P is *NEL-free* (short for *non-epiphenomenal libertarian free*) if and only if at least some of P's torn decisions are such that (a) they're TDW-undetermined (and hence also appropriately non-random and L-free), and (b) they're not inappropriately epiphenomenal—i.e., they play an appropriate role in the causation of P's actions.

More needs to be said about what it would mean for our torn decisions to be “inappropriately epiphenomenal.” The most obvious worry you might have about our torn decisions being epiphenomenal is based on the thought that (a) physical events always have physical causes, and so (b) our torn decisions can't cause *any* physical events (including bodily movements), because (c) torn decisions are *mental* events, not physical events. But I'm assuming mind-brain materialism here, and so while it's true that torn decisions are mental events, on the view I'm articulating, they're also physical events, presumably neural events. So this first worry doesn't even get off the ground.

But there's another worry you might have about our torn decisions being epiphenomenal. You might worry that (a) there are wholly non-conscious neural events that occur before our torn decisions that are common causes of our torn decisions and the corresponding actions, and (b) our torn decisions aren't causally upstream from our actions in the right way. In other words, you might worry that the causal map looks like this:

$$\text{Prior-to-conscious-choice neural events}\begin{array}{l} ↗\text{Conscious decision}\\ ↘\text{Action} \end{array}$$

If this is how things work in our brains, then it would seem to be freedom-undermining in an obvious sort of way. Thus, I'll assume that this is the relevant worry about our torn decisions being epiphenomenal. And so I'll take clause (b) of the definition of NEL-freedom to say that the torn decisions in question are not epiphenomenal in this way.

### 3.7. NE-Libertarianism

Given all this, we can say that *NE-libertarianism* is the view that human beings are NEL-free. In other words, it's the view that at least some of our torn decisions are (a) TDW-undetermined (and, hence, L-free) and (b) not epiphenomenal in the above way.

## 4. Responses to the Worries About Free Will

NE-libertarianism has a backward looking claim (namely, TDW-indeterminism) and a forward-looking claim (non-epiphenomenalism). These are both empirical claims, and so NE-libertarianism could be undermined by empirical findings that suggested that one or both of its empirical claims aren't true. The question I now want to ask is whether the empirical considerations discussed in section 2—i.e., F1-F5 and B1-B5—give us reason to think that NE-libertarianism isn't true.

I want to argue that the answer to this question is “No.” Indeed, now that we've got a clear picture of the sort of indeterministic, non-epiphenomenal freedom that we should be focused on—namely, NEL-freedom—I think it's easy to see that most of the supposedly anti-free-will considerations that I listed in section 2 are in fact entirely irrelevant to the question of whether human beings are NEL-free. In particular, it seems to me that all five of the forward-looking (epiphenomenalism-based) worries about free will from section 2 (i.e., F1-F5), and the first three of the backward-looking (determinism-based) worries (i.e., B1-B3), are *transparently* irrelevant to the question of whether we're NEL-free. In other words, the only anti-free-will considerations that I discussed in section 2 that aren't transparently irrelevant to the question of whether we're NEL-free are B4 and B5—i.e., the considerations based on the Libet studies and the Haynes studies. I'll discuss those studies in sections 4.2 and 4.3. For now, I just want to discuss F1-F5 and B1-B3.

### 4.1. F1-F5 and B1-B3

To illustrate the fact that considerations F1-F5 and B1-B3 are irrelevant to NE-libertarianism—i.e., to the thesis that we're NEL-free—I simply want to point out that NE-libertarianism is perfectly compatible with all of the following claims (NE-libertarianism doesn't entail any of these claims, but it's perfectly consistent with them):

1. The vast majority of our actions are not caused by—or, indeed, even preceded by—conscious choices. For example, when I take the 43rd step on my stroll through the park, I do not *decide* to do that in any interesting sense of the term; and the same thing is true of the vast majority of my actions.
2. We often have no idea why we do what we do.
3. We often have to infer what our reasons were for some of the actions we perform.
4. We often confabulate reasons for our actions, after the fact.
5. Many of our actions aren't caused by reasons at all—we just *do* them.
6. Conscious awareness of action often lags behind action—e.g., in speech processing and emergency situations.
7. We can sometimes be duped into thinking that we performed actions that we didn't perform; and we can sometimes be duped into thinking that we didn't perform actions that we did perform.
8. We are not directly aware of the causal link between our decisions and our actions; the claim that there's a causal link here is an empirical claim that requires evidence.
9. We do not have any good non-empirical reason to believe that our torn decisions are TDW-undetermined; indeed, for all we know, it could be that all of our torn decisions are fully determined by events that took place before we were born; the claim that TDW-indeterminism is true—i.e., that some of our torn decisions are TDW-undetermined—is an extremely controversial empirical hypothesis that requires evidence.
10. Many of our actions (and, indeed, many of our torn decisions) are causally influenced by subconscious mental states (and non-mental neural events) that we're not aware of at all.
11. Many of our actions (and, indeed, many of our torn decisions) are causally influenced by situational factors like mood.
12. Our torn decisions can be manipulated by external stimuli, e.g., magnetic stimulation to the brain. (Even if we assume that torn decisions can be causally influenced by magnetic stimulation to the brain, it doesn't follow that *ordinary* torn decisions—*without* magnetic stimulation—aren't TDW-undetermined. Here's an analogy: even if we can weight a coin to make it extremely likely that it will come up *heads* when we toss it, it doesn't follow that the outcomes of *fair* coin tosses are determined by prior events; it could be that the objective probability of getting *heads* on a fair coin toss is usually about 0.5. Or again: even if our torn decisions can be influenced by alien manipulation, it doesn't follow that when aliens aren't present, our torn decisions aren't TDW-undetermined and L-free).

All of these claims are perfectly compatible All of these claims are perfectlywith NE-libertarianism. This is entirely obvious—there's simply nothing in NE-libertarianism that says anything that's even remotely incompatible with any of the above claims. But the whole point of F1-F5 and B1-B3—i.e., the five forward-looking anti-free-will considerations and the first three backward-looking anti-free-will considerations—was that claims like the above (i.e., claims 1-12) are true. Thus, considerations F1-F5 and B1-B3 are all entirely irrelevant to the question of whether NE-libertarianism is true—i.e., whether we humans are NEL-free.

In a nutshell, the reason that F1-F5 and B1-B3 don't do anything to undermine NE-libertarianism—i.e., the reason that claims 1-12 are compatible with NE-libertarianism—is that (a) NE-libertarianism is a claim about *torn decisions only*, and (b) NE-libertarianism only says that *some* of our torn decisions are TDW-undetermined and non-epiphenomenal. If we keep these two points in mind when we read through claims 1-12, it becomes very clear that there's nothing in any of these claims that's incompatible with NE-libertarianism. Moreover, it also becomes clear that the anti-free-will argument here—the one based on claims like 1-12, or considerations like F1-F5 and B1-B3—is a straw-man argument. It's directed against a bizarre view of human beings that no one could take seriously. The NE-libertarian that I have in mind wants to respond to this argument by saying something like the following:

We're not idiots. We don't think that human beings are ideal (or event close to ideal) agents. We, of course, think that human beings are sometimes causally influenced by subconscious mental states and non-conscious brain processes that they're not aware of; and we, of course, think that human beings are often completely in the dark about why they do lots of what they do; and likewise for all of the claims that you're making here about human beings—we don't need to deny any of these claims. All we're saying—all that needs to be true in order for human beings to be NEL-free—is that *at least some or our torn decisions are TDW-undetermined and non-epiphenomenal*. And this is perfectly compatible with claims 1-12 and considerations F1-F5 and B1-B3.

It's important to note here that NE-libertarians can admit that *some* of our torn decisions are causally determined by factors that we're completely unaware of. Indeed, it seems to me that we have strong empirical reasons to believe that *many* of our torn decisions are causally influenced by factors that we're not aware of. But as far as I can see, we don't have any good reason to think that *all* of our torn decisions are causally influenced by such factors. To bring this out, consider an ordinary case in which an ordinary person—say, Ralph—makes a torn decision to order chocolate ice cream instead of vanilla. Do considerations like F1-F5 and B1-B3 give us good reason to think that *this* decision—made very calmly and consciously—wasn't TDW-undetermined and non-epiphenomenal? It seems to me that the answer to this question is obviously “No.” And it seems even more obvious that these considerations don't give us any good reason to think that *none* of our torn decisions is TDW-undetermined and non-epiphenomenal. The evidence we have just doesn't support this claim. Think of a typical day; you might make torn decisions about whether to have fruit or toast for breakfast, whether to take a walk before going to work, whether to work through lunch or go out to a restaurant, whether to work late or go to a concert, and so on. Does the existing evidence (in particular, the evidence concerning considerations like F1-F5 and B1-B3) really support the claim that *none* of these decisions is TDW-undetermined and non-epiphenomenal? The answer, I think, is that it does nothing of the sort. It supports the claim that we're often influenced by subconscious factors; but it just doesn't support the claim that *none* of our torn decisions is TDW-undetermined and non-epiphenomenal. Indeed, the existing evidence seems perfectly consistent with the thesis that a significant percentage of our torn decisions are TDW-undetermined and non-epiphenomenal. And that's all that NE-libertarians need^8^

At this point, you might object as follows:

You're not appreciating the fact that when we discover something about the way the mind-brain works in specific cases, we can infer that it works that way in *all* cases. So, for example, if consciousness is sluggish in some cases, then it's presumably sluggish in all cases. After all, it's not as if the neural processes involved in our conscious thinking can suddenly *speed up*.

I want to say two things in response to this objection, one related to the fact that (a) NE-libertarians think that we need to focus on *torn decisions in particular*, and one related to the fact that (b) NE-libertarians claim only that *some* of our torn decisions are TDW-undetermined and L-free. Point (a) is enough to give us a response to the worry about the sluggishness of consciousness. NE-libertarians obviously don't think that the neural processes involved in our conscious thinking sometimes speed up; rather, their position is that these processes don't *need* to speed up in order to be causally relevant to our torn decisions in the manner required for the truth of NE-libertarianism. Why? Because torn decisions are very different from, e.g., the processing of incoming speech and the jerking of steering wheels in emergency situations. Consciousness can't keep up with things like speech processing and emergency steering maneuvers; but there's no reason to think that it can't keep up with torn decisions. And this isn't because consciousness can “go faster” in connection with torn decisions; it's because there's no reason to think that torn decisions (about things like whether to order chocolate or vanilla ice cream) occur as quickly as speech processing and emergency steering maneuvers do. So we can't infer from the fact that consciousness is too sluggish to play a causal role in speech processing and emergency steering maneuvers to the conclusion that consciousness is too sluggish to play a causal role in torn decisions. So it's not that NE-libertarians are failing to take note of the fact that results obtained about specific cases generalize to other cases; it's rather that NE-libertarians are pointing out that the generalizing inference doesn't go through in the specific case at issue here because there are relevant disanalogies between torn decisions and, e.g., speech processing and emergency steering maneuvers.

Analogous points can be made about many of the other empirical results at issue in connection with considerations F1-F5 and B1-B3. But there's a second point that NE-libertarians need to make in order to provide a full response to the above objection. The second point concerns the psychology of our torn decisions rather than the neural processes involved in those decisions. The point is this: (a) there's no good reason to think that if some of our torn decisions are causally influenced by subconscious mental states or events (in ways that are incompatible with TDW-indeterminism), then *all* of them are; and (b) the sum total of the evidence that we presently have does not justify an inference to a claim of universality here. Now, I am not claiming that we could never be in position to infer from individual cases to a universal claim here. If we had the ability to locate torn decisions in our brains and to observe the causal antecedents of those decisions—and these are obviously things that we can't do right now—then if we observed a random (and reasonably large) sample of ordinary torn decisions and found that in all observed cases, our torn decisions were causally influenced by subconscious mental states or events (in ways that were incompatible with TDW-indeterminism), then it would be very rational for us to conclude that this was true in general. And so it would be rational for us to conclude in this scenario that NE-libertarianism was false. But we're just not in this situation right now. We don't have the ability to look at a random sample of ordinary torn decisions and determine whether they're causally influenced by subconscious mental states or events (in ways that are incompatible with TDW-indeterminism). And so while we've got good reason to think that *some* of our torn decisions are causally influenced by subconscious mental states or events, we're just not in a position to rationally infer that *all* of them are.

### 4.2. B4—The Libet Studies

Perhaps the most famous arguments against free will that have been generated by work in psychology and neuroscience are based on the work of Benjamin Libet. In this subsection, I'll explain why Libet's results don't give us any good reason to doubt NE-libertarianism—i.e., why they don't give us good reason to doubt that we're NEL-free.

Libet's studies were a follow-up to a neuroscientific discovery from the 1960s, in particular, the discovery that voluntary decisions are associated with a certain kind of brain activity known as the *readiness potential* (see e.g., Kornhuber and Deecke, 1965). Libet's studies were designed to determine a timeline for the readiness potential, the conscious intention to act, and the act itself (see e.g., Libet et al., 1983). In the main experiment, subjects sat facing a large clock that could measure time in ms, and they were told to flick their wrists whenever they felt an urge to do so and to note the exact time that they felt the conscious urge to move. What Libet found was that the readiness potential—the physical brain activity associated with our decisions—arose about 350–400 ms before the conscious intention to act and about 550 ms before the act itself. These results were immediately seen as raising a problem for free will. The argument against free will proceeds differently depending on the kind of free will that we have in mind. In our case, we can see Libet's results as raising a problem for TDW-indeterminism. In particular, the idea here is that (a) TDW-indeterminism requires indeterminacy at the moment of conscious choice, but (b) the fact that our conscious decisions are preceded by nonconscious brain processes (namely, the readiness potential) seems to suggest that the neural mechanisms responsible for our decisions are already up and running before our conscious thinking enters the picture.

The problem with this reasoning is that it's not clear what the *function* of the readiness potential is. In particular, there is no evidence for the claim that, in torn decisions, the readiness potential is causally relevant to which option is chosen^9^ There are many other things that the readiness potential could be doing. One way to see that this is true is to recall from section 3 that NE-libertarianism is perfectly consistent with the idea that various aspects of our torn decisions are causally determined. In particular, as we saw above, a torn decision could be TDW-undetermined and NEL-free even if it was determined in advance that (i) the torn decision in question was going to occur, and (ii) the choice was going to come from among the agent's tied-for-best options, and (iii) the objective moment-of-choice probabilities of these options being chosen were all more or less even. The only thing that needs to be undetermined, in order for a torn decision to be TDW-undetermined and NEL-free, is *which tied-for-best option is chosen*. Given this, it should be obvious how NE-libertarians can respond to the Libet studies. They can say that for all we know, it could be that the readiness potential is part of a process that's causally relevant to our torn decisions but doesn't causally influence which tied-for-best option is chosen. For instance, it could be part of a causal process that leads to the *occurrence* of a torn decision without influencing which tied-for-best option is chosen^10^ Or it could be that the readiness potential is part of the process whereby our reasons cause our decisions; and it could be that while in connection with certain kinds of non-torn decisions this process determines which option is chosen, in connection with torn decisions, it merely causes the choice to come from the agent's tied-for-best options (and perhaps also causes the objective moment-of-choice probabilities of these options being chosen to be more or less even).

So the point here is that we don't presently have good reason to think that, in torn decisions, the readiness potential is causally relevant to which tied-for-best option is chosen. There just isn't any evidence for this, and so the existence of the readiness potential gives us no reason to think that, in torn decisions, which tied-for-best option is chosen is causally affected by prior-to-choice nonconscious brain processes. So it doesn't give us any good reason to doubt TDW-indeterminism. In other words, the existence of the readiness potential is perfectly compatible with the NE-libertarian claim that some of our torn decisions are TDW-undetermined^11^

### 4.3. B5—The Haynes Studies

I now want to consider the objection to NE-libertarianism that's based on Haynes's studies. *Prima facie*, these studies seem to give rise to a devastating objection to TDW-indeterminism, but I'm going to argue that this appearance is deceiving and that, in fact, Haynes's studies don't give us any good reason to doubt TDW-indeterminism.

Haynes's studies seem tailor-made to provide anti-libertarians with a way of responding to what I just said in section 4.2 about the argument based on Libet's studies. My central objection to that argument was that it fails to distinguish between the *occurrence* of a torn decision and the issue of *which tied-for-best option is chosen*. More specifically, my objection was that for all we know right now, the readiness potential could be part of what causes our torn decisions to occur without doing anything to cause a specific tied-for-best option to be chosen. But Haynes's studies seem to be explicitly constructed to block this sort of response. To bring this out, let's recall how the main Haynes study went. Haynes gave his subjects two buttons, one for the left hand and one for the right, and he told them to make a decision at some point as to which button to push, and he used a very simple method to estimate the time at which the conscious decision occurred (in particular, subjects were presented with a randomized stream of letters, and they had to report which letters they were looking at when they made their conscious decisions). What Haynes found was that there was unconscious neural activity in two different regions of the brain that predicted whether subjects were going to press the left button or the right button. Moreover, he found that this activity arose as long as 7–10 s before the person's conscious decision to push the given button.

These results seem to generate a serious objection to TDW-indeterminism and NE-libertarianism. For (a) the results seem to suggest that our decisions are already determined before we make them, and (b) TDW-indeterminacy (and NEL-freedom) require indeterminacy at the moment of conscious choice. *Prima facie*, this line of thought seems extremely powerful, but I want to argue that when we look at the details of Haynes's study, the argument against TDW-indeterminism completely falls apart.

There are two details of the study that I want to discuss. The first has to do with the specific *regions* of the brain where the pre-conscious-choice neural activity was found; in particular, it was found in the *parietal cortex* (or for short, PC) and in what's known as *Brodmann area 10* (or for short, BA10). Why this is important will become clear below. The second important detail is this: the pre-choice brain activity that Haynes found (in PC and BA10 regions) was actually not a very reliable guide to predicting the outcomes of his subjects' choices. Indeed, it was only 10% more reliable than blind guessing. If we just guess which button subjects are going to push, we'll be right about 50% of the time, whereas if we use information about the activity in PC and BA10 regions of subjects' brains, we'll be right at best 60% of the time. This is definitely statistically significant, so it's showing *something*. But it's not immediately obvious *what* it's showing, and as I will explain in what follows, it *doesn't* show (or, indeed, give us any good reason to believe) that TDW-indeterminism and NE-libertarianism are false.

But let me slow down and explain the significance of the fact that the pre-choice brain activity was found in PC and BA10 regions of the brain. The strange thing about this is that these regions are not associated with free conscious decisions. However, they *are* associated with *plans*, or *intentions*. In particular, they're associated with the generation and storage of plans^12^^,^^13^ This is extremely important. In fact, when we combine this with the fact that the neural activity in PC and BA10 regions is only 10% more predictive than blind guessing, the argument against TDW-indeterminism comes unraveled. The reason is that when we put these two facts together, they suggest an alternative explanation of Haynes's results that's perfectly consistent with TDW-indeterminism and NE-libertarianism. I will say in a moment what this alternative explanation is, but before I do, I need to make a background point.

When someone asks you *not* to think about something, it suddenly becomes very difficult to obey them. For instance, if I don't want you to think about Abraham Lincoln right now, one of the worst things I could do is *tell* you not to think about him. If I just say nothing, then the odds that you would think of Lincoln in the next few minutes are vanishingly small. But as soon as I say, “Don't think about Abe Lincoln,” it becomes very hard for you to avoid thinking about him, even if you sincerely want to obey me. The problem is that the temptation to think about what you're not supposed to think about can be almost overwhelming.

The same goes for little *decisions*, like picking a number between 1 and 10. Suppose I say this to you: “In a minute, I'm going to ask you to pick a number between 1 and 10, but don't do it yet.” It's actually rather difficult to refrain from thinking of a number in situations like this. Indeed, it's fairly likely that before I can even spit out the second half of my sentence, you will already have thought of a number between 1 and 10. As soon as I tell you that you're going to be asked to pick a number between 1 and 10, you might pick the number 7 before you even hear me say that you shouldn't choose yet.

Now, once you hear me tell you that you're not supposed to pick yet, you might try to undo what you already did—i.e., you might try to unpick the number 7. But the result of this will probably not be that 7 gets, so to speak, “put back into the hopper.” Instead, it will be that 7 is eliminated from consideration all together. This is because we can't turn ourselves into random number generators. The problem is that you won't be able to forget that you already thought of the number 7. So after a minute passes and I tell you to pick a number, it's unlikely that you'll pick 7 again. If you did, you wouldn't think you were being truly random and that it was just a coincidence that you picked 7 twice in a row; you'd probably think you were *cheating*—that you were flagrantly disobeying the command not to choose in advance. So even if you didn't realize this, I think the real result of undoing your choice would very likely be that 7 is simply eliminated from consideration.

But now suppose that instead of telling you that you're going to have to pick a number between 1 and 10, I tell you that you're going to have to pick either the number 1 or the number *2*. And suppose that you instantly think of the number 2. Now, what's going to happen when I tell you that I don't want you to choose yet, that I want you to wait 60s and *then* pick a number? You might try to unpick the number 2, but if the result of this is that 2 is eliminated from consideration, then the only option left is 1. So unless you really manage to completely forget about the fact that you chose the number 2 before, the choice you end up making is not going to be truly random. It's going to be weirdly influenced by your attempt to follow the instructions despite the fact that you started off by picking the number 2.

So that's one point. Here's another point: even if you don't start out by thinking of one of the two numbers, it's actually somewhat difficult to keep yourself from thinking of one of them. Try it right now. Flip an hourglass over and tell yourself that you're not going to think of 1 or 2 until all the sand runs out and that, when the sand does run out, you're going to choose one of the two numbers. This isn't that easy. I'm not saying you *can't* succeed in doing it. Of course you can. You might be able to distract yourself and think about something else entirely. But you might *not* succeed. In short, the point here is that *sometimes*, when we're asked not to think about something, we fail.

Now, here's the really important point for us. You might fail in this task *even if you don't realize it*. You might subconsciously think of the number 1, and you might subconsciously store the plan to pick that number when the time comes. This shouldn't be controversial at all. For here are two things that we know to be true about humans: first, it's somewhat difficult for us to avoid thinking about something when someone tells us not to think about it; and second, we do all sorts of things unconsciously. We might not do *everything* unconsciously, but it's clear that we do a *lot* of things unconsciously. When we put these two points together, we get the following (highly probable) hypothesis:

If you tell a group of human subjects that in 60s they're going to be asked to pick the number 1 or the number 2, and if you tell them not to pick yet—in other words, if you tell them to wait until the 60s are up before they choose—at least *some* of these subjects will (without realizing it) subconsciously think of one of the two numbers before the 60s have elapsed, and they will subconsciously store the plan to pick that number when the time comes.

Again, given what we know about ourselves, this seems extremely plausible. Indeed, it seems to me that it would be surprising if it *wasn't* true. (By the way, I'm not claiming here that any time someone subconsciously *thinks* of a given option, she *commits* to it. That's obviously not true. But all that's needed here—and this will come out more clearly below—is that in cases like the ones we're considering here, there can be subconscious mental activity that causally influences how the decisions go. And, again, this doesn't seem very controversial.)

In any event, this is all just background. But it's highly relevant to the Haynes studies because it suggests an explanation of Haynes's results that's perfectly consistent with TDW-indeterminism and NE-libertarianism. The explanation that I have in mind—and we'll see later that this isn't the only explanation of Haynes's results that's compatible with TDW-indeterminism and NE-libertarianism—can be put in the following way:

*An explanation of Haynes's results that's perfectly consistent with TDW-indeterminism and NE-libertarianism:* A significant percentage of the subjects in Haynes's study (say, 20% of them) unconsciously failed to make truly spontaneous decisions about whether to press the right button or the left button. They genuinely *wanted* to follow Haynes's instructions, but for whatever reason, and without realizing it, they unconsciously formed prior-to-choice plans to push one of the two buttons. They unconsciously stored this information in their brains, and then when the time came, these plans were activated. In other words, the regions of the brain where these plans were stored were activated. And this brain activity caused the subjects to choose in the ways in which they had unconsciously planned on choosing. This explains why (in *some* subjects) there was prior-to-choice brain activity in PC and BA10 regions of the brain (and, remember, while these regions are associated with the formation and storage of *plans*, they're *not* associated with free conscious decisions). It also explains why this brain activity predicts whether subjects will push the left button or the right button. And finally, it also explains why using this brain activity to predict how subjects will choose is only 10% more reliable than blind guessing—the reason is that *not all* subjects unconsciously formed plans about what they were going to do. Only *some* of them did. Most of the subjects managed to avoid doing this, and so most of them succeeded in making truly spontaneous decisions. (Of course, the claim here isn't that most of us are NEL-free, but some of us aren't. The claim is that *all* of us *sometimes* fail to be NEL-free; we're all *sometimes* driven by things like unconscious plans; but we aren't *always* driven by such things.)

The first point to note about this explanation is that if it's right, then there's no problem here for TDW-indeterminism or NEL-freedom. All Haynes's results show is that sometimes our decisions are influenced by unconscious factors. But we already *knew* this. NE-libertarians don't think (or at any rate, they shouldn't think) that all of our torn decisions are NEL-free. As we've already seen, they should admit that our torn decisions are often causally influenced by unconscious factors in ways that make it the case that they're not TDW-undetermined. What NE-libertarians claim is that this isn't *always* the case—i.e., that *some* of our torn decisions are TDW-undetermined. But given this, if my explanation of Haynes's findings is correct, then those findings don't give us any good reason to doubt the NE-libertarian view because they don't give us any good reason to think that our torn decisions are never TDW-undetermined. All they show is that our torn decisions aren't *always* TDW-undetermined. And so these findings are perfectly consistent with the NE-libertarian view that *some* of our torn decisions are TDW-undetermined and NEL-free.

One might object to my argument here in something like the following way:

Whenever someone uses scientific data to argue for a hypothesis H, we can always respond to the argument by presenting an alternative explanation of the data that doesn't involve the claim that H is true. But in order to have a *good* response to the argument for H, the alternative explanation can't be a cockamamie story. It has to be just as plausible (or just as likely to be true) as the original explanation—i.e., the explanation that leads to the conclusion that H is true. But in our case, it's not clear that your alternative explanation of Haynes's findings is as plausible as explanations that are hostile to TDW-indeterminism and NE-libertarianism.

I want to respond to this objection by arguing that my explanation is actually *more* plausible—or more likely to be true—than any explanation that's hostile to TDW-indeterminism and NE-libertarianism. In order to argue for this, I first need to clarify what these other explanations (that are hostile to TDW-indeterminism and NE-libertarianism) *say*. There are two different views that enemies of TDW-indeterminism might endorse here, namely, the following:

*The early-signature-of-the-decision view*: The brain events that Haynes found (in PC and BA10 regions of the brain) were early neural signatures of the conscious decisions themselves—i.e., the decisions that the subjects experienced 7–10 s later.*The prior-cause view*: The brain events that Haynes found occurred prior to the subjects' conscious decisions, and they caused those decisions to go in the ways that they went.

But there are problems with both of these views—or at any rate, with opponents of TDW-indeterminism endorsing these views. Let me start with the prior-cause view. The first point I want to note about this view is that, as it's stated here, it's compatible with TDW-indeterminism. Indeed, the interpretation of Haynes's results that I'm proposing in this paper more or less *entails* the prior-cause view—for according to that interpretation, the outcomes of Haynes's subjects' conscious decisions were caused by events in which the subjects subconsciously formed prior-to-conscious-choice plans to choose in certain ways. But it's crucial to this interpretation that this is true of only *some* of Haynes's subjects; in other words, according to the interpretation I'm proposing, Haynes's results don't give us good reason to think that this generalizes to *all* subjects.

It's worth pausing to emphasize the sort of TDW-indeterminist/NE-libertarian view we're talking about here. Before we even encountered Haynes's studies, we already acknowledged that TDW-indeterminists (and NE-libertarians) would be wise to admit that *some* of our torn decisions are causally determined by subconscious mental states or events. And these theorists were already committed to claiming that as of right now, we don't have any reason to think that this is *universally* true. We can think of the interpretation of Haynes's results that I'm proposing here along these lines. What I'm suggesting is that TDW-indeterminists can say this:

Look, we already admitted that *some* of our torn decisions are causally influenced by prior events (and, hence, that some of these decisions are not TDW-undetermined). Haynes's results just confirm this point.

So if you want to claim that Haynes's results undermine TDW-indeterminism, and if you want to endorse the prior-cause view, then you need to endorse the following:

*Allism*: Haynes's results suggest that the outcomes of *all human torn decisions* are caused by prior-to-conscious-choice brain events.

But it's hard to see how we have any reason to believe this. If what I argued in previous sections of this paper is right, then before Haynes performed his studies, there was a plausible view on the table according to which *some but not all* of our torn decisions are causally determined by prior events. (This view is obviously controversial; my claim is just that, prior to Haynes's study, it was compatible with our evidence.) But given this, it seems that in order for us to have good reason to believe allism—i.e., in order for us to plausibly claim that Haynes's results show that *all* of our torn decisions are causally determined by prior events—we would need evidence for the claim that causal factors of the kind that Haynes found occur in all cases. But we just *don't* have evidence for this. For all we know, it could be that causal factors of the kind that Haynes found are present in some cases not but not all. For example, it could be that the causal factors that Haynes found have to do with the causation of torn decisions by subconscious mental states or events, and it could be that while this kind of causation is present in some cases, it's not present in all cases. More generally, the claim that I'm making here is that (i) we don't have any good reason to think that all torn decisions are caused by events of the same kind, and (ii) Haynes's results don't do anything to change this situation.

These remarks bring out an important point. There's nothing special about the interpretation of Haynes's results that I'm suggesting here—i.e., the interpretation that has to do with the formation of subconscious plans. This is just one interpretation among many that TDW-indeterminists could endorse. All that TDW-indeterminists need to say here, in order to maintain that Haynes's results don't undermine their view, is this: while Haynes's results do seem to suggest that our torn decisions are sometimes caused by prior events, there's no evidence for the claim that the causal factors that Haynes has found are present in all cases. My story about subconscious plans is one story of the some-but-not-all kind that TDW-indeterminists can tell here; but it's not the only one.

So I don't think the prior-cause view gives us a plausible way of attacking TDW-indeterminism. What about the early-signature-of-the-decision view? Well, one thing this view has going for it is that it avoids the problem I just raised for the prior-cause view. For while we don't have any good reason to think that all of our torn decisions are caused by events of the same kind, I think that we do have good reason (at least until we're proven wrong) to suppose that torn decisions are neural events of a fairly unified kind. So if observation revealed that some torn decisions were neural events of some kind K, that would give us *prima facie* reason to think that other torn decisions were of that kind.

But I don't think the early-signature-of-the-decision view is very plausible. There are at least three different arguments for thinking that my explanation of Haynes's data is more plausible than the early-signature-of-the-decision view. Here are the three arguments:

1. We have strong independent evidence for the hypothesis that PC and BA10 regions of the brain are relevant to the formation and storage of plans and intentions, and we have no reason to think that these regions are relevant to conscious decisions. Therefore, since my explanation takes the brain activity that Haynes found in those regions to be related to the formation and storage of long-term plans, it fits with what we already know about those regions, and so it's more plausible than the early-signature-of-the-decision explanation, which takes this activity to be an early neural signature of the conscious decision itself.
2. The fact that there's a 7–10 s time gap between the brain activity in PC and BA10 regions and the conscious decision counts as strong evidence that that brain activity is *not* part of the decision. This is a bit ironic because, intuitively, the 7–10 s gap is the thing that makes Haynes's results so striking. When you first hear about these studies, you're likely to think that if neuroscientists can predict how you'll choose 7–10 s before you make a conscious decision, then you couldn't possibly be NEL-free. But upon further reflection, the 7–10 s time gap turns out to be part of what *undoes* the Haynes argument. This is because we have extremely strong reasons to think that human beings are way faster than this when it comes to making decisions. There is experimental evidence (see e.g., Trevena and Miller, 2010) that suggests that we can make decisions in *less than half a second*. Moreover, we all *know* that this is true. We have all had lots of experience making snap decisions in way less than 7 s. Therefore, since we know that decisions take less than 7 s, it's not plausible that the brain activity that Haynes observed—a full 7-10 s before the conscious choice—was an early neural signature of the conscious decision itself. It's much more plausible to suppose that this brain activity was doing something else. And my explanation provides a compelling story about what it was doing—it was related to the storage of a long-term plan that was made unconsciously and unwittingly by the subject.
3. My interpretation of the data explains why using the brain activity in PC and BA10 regions is only 10% more reliable than blind guessing. It's because only *some* of the subjects unwittingly formed unconscious plans about what they were going to do. Some of them didn't do this. Some of them managed to refrain from doing this so that their conscious decisions were genuinely spontaneous last-second choices. On the other hand, the early-signature-of-the-decision explanation of Haynes's results *doesn't* explain why using the brain activity in PC and BA10 regions is only 10% more reliable than blind guessing. People who favor the early-signature-of-the-decision explanation have no option but to say that the reason there's only a 10% increase in reliability here is that we're just not good enough yet at gathering data from people's brains. This seems much less plausible to me.

So, again, it seems to me that my explanation of the data is better than the early-signature-of-the-decision explanation. Now, I don't want to claim that I've proven that the latter explanation is definitely wrong. It is, of course, *possible* that the brain activity in PC and BA10 regions *is* an early neural signature of the conscious decision itself. But there's no *evidence* for this. Thus, it seems to me fair to conclude that Haynes's results don't give us any good reason to doubt the NE-libertarian hypothesis that some of our torn decisions are TDW-undetermined and NEL-free^14^^,^^15^

In closing, I should say that I do not take myself to have provided a positive argument for NE-libertarianism, and in fact, I don't think we have any very good reason to believe it. But I also think that we don't have any good reason to *dis*believe it.

## Author Contributions

The author confirms being the sole contributor of this work and has approved it for publication.

### Conflict of Interest Statement

The author declares that the research was conducted in the absence of any commercial or financial relationships that could be construed as a potential conflict of interest.

## Footnotes

^1^I suppose you might think that just as compatibilists reject (D2), so too we should reject (E2). Now, my own view is that the idea that (E2) is false (i.e., that free will is compatible with epiphenomenalism) is considerably less plausible than the idea that (D2) is false (i.e., that free will is compatible with determinism). But it doesn't matter; for I would deal with the suggestion that (E2) might be false in the same way that I'm dealing here with the suggestion that (D2) might be false, namely, by just stipulating that I'm talking about a non-epiphenomenal kind of freedom—i.e., a kind of freedom that just *is* incompatible with epiphenomenalism.

^2^Many people have defended libertarian views. Recent examples include van Inwagen (1983), Ginet (1990), Clarke (1993), Kane (1996), Ekstrom (2000), O'Connor (2000), Griffith (2007), Balaguer (2010), Franklin (2011), Mawson (2011), Steward (2012), and Todd (2016).

^3^I should say that it's *possible* to make a torn decision while in a Buridan's-ass situation—because you could be weird enough to *care* which can of Campbell's tomato soup you get, and so you could feel genuinely torn about it. But *most* of us don't make torn decisions in Buridan situations. For example, in the above situation, most of us would just grab a can of soup without thinking about it.

^4^Again, to be more precise, I should say that libertarianism is compatible with the claim that some of our torn decisions are determined by *events involving* us having subconscious reasons that we're not aware of. I won't keep making this clarification.

^5^I guess there's a usage of the term “determined” on which expressions like ‘partially determined' don't make sense. But I'm not using the term in that way.

^6^Arguments of this general kind have been put forward many times by numerous philosophers. See, e.g., Hobbes (1651), Hume (1748), Hobart (1934), Fischer (1999), Haji (1999), Mele (1999), and Levy (2011).

^7^TDW-indeterminism implies that nothing causally influences the decision at the moment of choice; so it guarantees that the decision is itself an undetermined event—indeed, *the* undetermined event that determines whether I get chocolate or vanilla ice cream. It might seem that the indeterminacy could be resolved by an event that occurs before the decision; but the assumption of TDW-indeterminism rules out this possibility because it requires indeterminacy *at the moment of choice*.

^8^Strictly speaking, all NE-libertarianism says is that *some* of our torn decisions are NEL-free. But it's plausible to suppose that there's a good deal of regularity here, so that if any of our torn decisions are NEL-free, then a *significant percentage* of them are—or some such thing.

^9^Indeed, we have good reason to think that the readiness potential is *not* part of a causal process that's relevant to which option is chosen. The lateralized readiness potential (LRP) is a more plausible candidate for being relevant here; for more on this, see Haggard and Eimer (1999) and Haggard's contribution to Haggard and Libet (2001); and for an argument that even the LRP isn't part of a causal process that's relevant to which option is chosen, see Schlegel et al. (2013).

^10^A similar point, though a bit different, has been made by Haggard and Eimer (1999); and, again, see also Haggard and Libet (2001).

^11^Responses to Libet-style worries about free will have been given by many people. See e.g., Mele (2009), Balaguer (2010), Bayne (2011), Roskies (2011), Schurger et al. (2012), Levy (2014), Nahmias (2015).

^12^For evidence that the BA10 region is associated with the storage of plans and intentions, see, e.g., Burgess et al. (2001), Haynes et al. (2007). And for evidence that the PC region is associated with the generation of plans, see e.g., Desmurget and Sirigu (2009).

^13^You might think that to make a decision just *is* to generate a plan [see e.g., Mele (2009) for a view along these lines]. I think there are problems with this definition, but it doesn't matter here. For instead of speaking of *decisions*, we can speak of *conscious* decisions. It may be that if I subconsciously generate a plan to do something then I've made a “decision” in some (I think pretty odd) sense of the term; but I certainly haven't made a conscious decision.

^14^There's another point worth making here that's pretty ironic. The early-signature-of-the-decision view doesn't actually undermine TDW-indeterminism. For if it were really true that the brain events that Haynes found were early neural signatures of the decision itself, then the proper conclusion to draw would be that the relevant brain events were *parts* of the conscious decision, not prior to it. But if they're parts of the decision, then there's no problem here for TDW-indeterminism. (I don't actually believe that these events are parts of the decision; but that's only because I don't believe that decisions take 10 s to occur; I'm simply pointing out what you should say if you do believe that decisions take 10 s to occur.)

^15^Other responses to Haynes-style worries about free will can be found in Balaguer (2014), Levy (2014).
